# Surgical Rodent Models for the Study of Peripheral Arterial Disease

**DOI:** 10.3390/biomedicines14061393

**Published:** 2026-06-20

**Authors:** Lauren Carmon, Kristopher Maier, Vivian Gahtan

**Affiliations:** 1 Department of Surgery, Loyola University of Chicago, Maywood, IL 60153, USA; lcarmon@luc.edu (L.C.); kmaier2@luc.edu (K.M.); 2 Research Service, Edward Hines Jr. VA Hospital, Hines, IL 60141, USA

**Keywords:** neointimal hyperplasia, intimal hyperplasia, arterial injury, angiogenesis, arteriogenesis, ischemia–reperfusion

## Abstract

Cardiovascular disease remains the leading cause of morbidity and mortality worldwide, with atherosclerosis and maladaptive vascular remodeling serving as central drivers of clinical events. Mechanistic investigation of arterial disease processes relies heavily on experimental animal models that permit precise control of vascular injury, hemodynamic forces, and ischemic stress. Over the past several decades, murine and rat models have become indispensable tools for studying endothelial dysfunction, intimal hyperplasia, flow-mediated remodeling, and ischemia–reperfusion injury. Each model reproduces distinct aspects of human vascular pathology while offering unique technical and biological advantages. This review summarizes commonly used murine and rat models of arterial disease, emphasizing the biological mechanisms they study, the surgical techniques used, pathophysiology, experimental endpoints, advantages, and limitations.

## 1. Introduction

Cardiovascular diseases are widely recognized as the leading cause of disease burden and mortality globally, leading to 19.2 million deaths in 2023 [1]. The cardiovascular disease burden continues to increase, driven by population aging, population growth, and the increasing prevalence of atherosclerotic cardiovascular disease [1]. Atherosclerosis underlies the majority of cardiovascular disease by causing progressive vascular occlusion, most commonly in the coronary vessels, carotid arteries, and peripheral vasculature [1,2]. The development of animal models of cardiovascular disease has provided us with crucial insights into the pathophysiology of various vascular diseases, ultimately facilitating the translation of basic scientific discoveries into effective clinical therapies. This review summarizes commonly used surgical rodent models of peripheral arterial disease (PAD), emphasizing the biological mechanisms they study, the surgical techniques used, pathophysiology, experimental endpoints, advantages, and limitations.

### Rodent Species and Models Used in Peripheral Arterial Disease Research

Mice and rats are the predominant species used in experimental PAD research due to their well-characterized genetics, reproducibility, relatively low cost, and amenability to surgical manipulation [3]. The C57BL/6 mouse is the most widely used inbred strain, serving as the genetic background for the majority of transgenic and knockout models used in flow-mediated remodeling and hindlimb ischemia research (Table 1) [4]. Sprague-Dawley and Wistar rats are frequently used strains for atherosclerosis and intimal hyperplasia (IH) research [2]. Additionally, rats that mimic certain aspects of cardiovascular pathology, including the spontaneously hypertensive rat, Dahl salt-sensitive rat, and Zucker diabetic fatty rat, can be used to study the influence of cardiovascular risk factors on IH (Table 1) [2,5,6]. These models, combined with surgical injury models, allow for translational research of cellular and molecular processes compared with solely mechanical injury models [2]. Similarly, transgenic or knockout strains enable the study of phenotypes of acquired or heritable metabolic disorders, providing complementary tools for investigating the initiation and progression of atherosclerotic vascular disease in combined injury or diet models (Table 1) [2,3]. Notably, Apolipoprotein E-deficient (ApoE−/−) mice and rats have been established as a reliable model, especially when combined with a high-fat diet, to investigate atherosclerosis initiation and progression (Table 1) [2,3,7,8].

## 2. Carotid Artery Balloon Injury Model

### 2.1. Background

Atherosclerosis is a chronic inflammatory disease characterized by plaque formation in medium and large arteries, leading to arterial narrowing or blockage, tissue loss, infection, and potential limb loss or even death [21]. To prevent limb loss, revascularization can be performed to open the stenotic area and restore blood flow to the obstructed vessels [21]. Unfortunately, restenosis secondary to uncontrolled IH, vascular remodeling, and vascular smooth muscle cell (VSMC) proliferation is a well-established limitation of reconstruction procedures, including angioplasty, endarterectomy, and bypass [22,23,24,25]. Restenosis, characterized by reduced vessel diameter and neointimal thickening, results from maladaptive vascular wound healing driven by endothelial denudation and medial injury [22]. IH occurs in up to 60% of patients within the first year after angioplasty, and vascular remodeling may account for 50–90% of late luminal area loss [23,24].

Because the arterial injury response is rarely available for study in humans, animal models, including the carotid artery balloon injury model, have been essential for elucidating the mechanisms underlying post-intervention vascular repair [22]. The carotid artery balloon injury model replicates the mechanical endothelial and medial injury characteristic of angioplasty and has therefore become a standard rat model to study vascular injury responses (e.g., VSMC proliferation, neointimal formation) and therapeutic strategies aimed at preventing restenosis [25].

### 2.2. Surgical Technique

**Figure 1:**
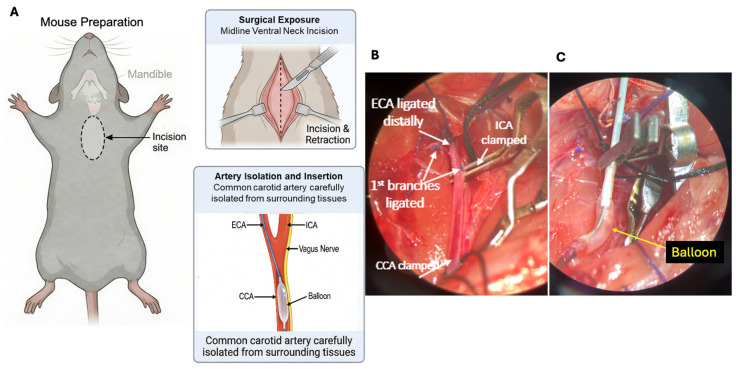
Carotid artery balloon injury model. (**A**) Schematic representation of the surgical approach. Animals are anesthetized and positioned supine, followed by a midline ventral neck incision. The tissue is retracted to the left, a deeper dissection ensues and the common carotid artery (CCA), internal carotid artery (ICA) and external carotid artery (ECA) are carefully isolated from surrounding connective tissue and adjacent structures, including the vagus nerve. Following vessel isolation, an arteriotomy is performed in the ECA to allow insertion of the balloon catheter into the CCA. (**B**) Intraoperative image demonstrating vascular control and preparation for balloon injury. The ECA is ligated distally, branch vessels are secured, and the ICA and the CCA are temporarily clamped to permit controlled catheter manipulation. (**C**) Representative image of balloon inflation within the CCA, producing endothelial denudation and controlled medial injury. Abbreviations: CCA: Common Carotid Artery; ECA: External Carotid Artery; ICA: Internal Carotid Artery.

Sprague-Dawley rats are most commonly utilized for the rat carotid artery balloon injury method [26]. Our convention is to use the left side for injury and to preserve the right side as a control vessel. The animal is placed lying supine after induction and is maintained in a surgical plane of anesthesia with isoflurane. Hair is removed from the incision site, and the area is sanitized with alcohol pads and betadine swabs. Surgery is performed using a dissecting microscope or surgical loupes for magnification [27]. A straight incision is made at the midline of the neck from immediately below the chin to the top of the sternum, and the skin is separated from the underlying glandular tissue (Figure 1) [26]. A fascial incision is then made, and the glands are separated using blunt dissection to expose the muscular layer. Sharp fine forceps are used to separate the muscular tissue to expose the left common carotid artery (CCA) and the vagus nerve [26]. Blunt dissection is continued distally to expose the carotid artery bifurcation into the internal carotid artery (ICA) and external carotid artery (ECA). Three 4-0 silk sutures are placed around proximal CCA, proximal ECA (just beyond the bifurcation) and distal as feasible within the dissection of the ECA to provide retraction and hemostasis. If arterial branching off the ECA is apparent, the ascending pharyngeal, occipital, and/or superior thyroid artery is/are tied off using 6-0 Prolene (Ethicon, Guaynabo, Puerto Rico) suture. Prior to making an arteriotomy, micro clamps are placed on the ICA and proximal CCA. Various techniques may be used to induce arterial injury. In our lab, a small transverse incision is made in the ECA and a 2F Fogarty thrombectomy balloon catheter (Edwards Lifesciences, Unterschleissheim, Germany) is inserted (Figure 1). The CCA clamp is removed. The balloon is then inflated with saline using an insufflator (5 atmospheres, 5 min), inducing arterial injury, which will lead to IH resulting in arterial narrowing. The balloon is deflated and removed. The ECA is ligated, the ICA clamp is removed, and flow is restored. The incision is closed with a 4-0 Vicryl (Ethicon, Guaynabo, Puerto Rico) suture.

Different techniques can be used to induce arterial injury [26]. A model of denudation and a dragging force can be used. The 2F Fogarty balloon is passed into the ECA and the clamp on the CCA is then removed, and the balloon catheter is advanced down the CCA to the aortic arch [26]. The balloon catheter is then inflated with a lower pressure than described above and slowly withdrawn with rotation, stopping short of the carotid bifurcation. The balloon catheter is then deflated, and the process is repeated. This process of inserting, inflating, retracting, and deflating the catheter should be performed a total of three times to ensure complete and reproducible removal of the endothelial lining and distention of the vessel wall [23,26].

Two methods for balloon inflation are used: a liquid-filled syringe or pressure-based balloon inflation [23]. Both methods enable the precise inflation of the balloon to a predetermined volume (suggested 0.02 mL) or pressure (~2.0 atm), thereby achieving reproducible injury [23,26]. One disadvantage of a liquid-filled syringe is the risk of air bubbles [28]. Therefore, more recent studies have suggested that pressure-based balloon inflation may be preferred over a liquid-filled syringe due to the reduced risk of air bubbles and variability in the pressure applied, and therefore variability in the injury itself [28]. Angioplasty balloons have also been used to induce injury, which have an equal radial force. The Fogarty balloon is compliant and with insufflation generally causes asymmetric force across the vessel.

Another method is wire denudation, where an angiocatheter guide wire is inserted into the carotid or femoral artery for 1 min. This technique results in arterial injury by denuding the endothelium and distending the vessel wall [29]. The wire is then removed, blood flow is restored, and the skin incision is closed [27].

### 2.3. Pathophysiology

Balloon catheter-induced vascular injury initiates a cascade of structural and cellular events that ultimately causes IH [22]. Mechanical inflation and withdrawal of the balloon denudes the endothelial lining, exposing the highly thrombogenic subendothelial matrix [22,29]. Thus, upon reperfusion, platelets rapidly adhere, activate, and degranulate, forming a platelet layer on the arterial wall [22]. Inflammatory cells, including neutrophils and macrophages, are also recruited to the injured vessel. These pro-inflammatory macrophages release cytokines and chemokines that drive VSMC migration from the media to the intima, resulting in VSMC differentiation from their native ‘contractile’ phenotype to a ‘synthetic’ form that comprises the majority of the neointima [29]. In addition to migration and differentiation, growth factors, including platelet-derived growth factor, stimulate VSMC proliferation [22,30]. These extracellular stimuli further activate downstream signaling pathways such as phospholipase C, protein kinase C, and/or proto-oncogenes, including c-fos, c-myc, and c-myb, which are also associated with VSMC proliferation [31].

In the intima, VSMCs continue to proliferate until the luminal surface is formed by a thick layer of VSMCs rather than by an endothelium, resulting in wall thickening, luminal narrowing, and ultimately gradual loss of vessel patency [22,29]. The ‘synthetic’ VSMCs also produce an extracellular matrix composed of collagen, elastin, and proteoglycans, which further increases intimal thickness, VSMC migration and proliferation, and immune cell adhesion [22,29]. The combined effects of inflammatory cell accumulation, VSMC proliferation and migration, and extracellular matrix deposition form a neointima that narrows the lumen and can lead to restenosis [22].

### 2.4. Experimental Endpoints

**Figure 2:**
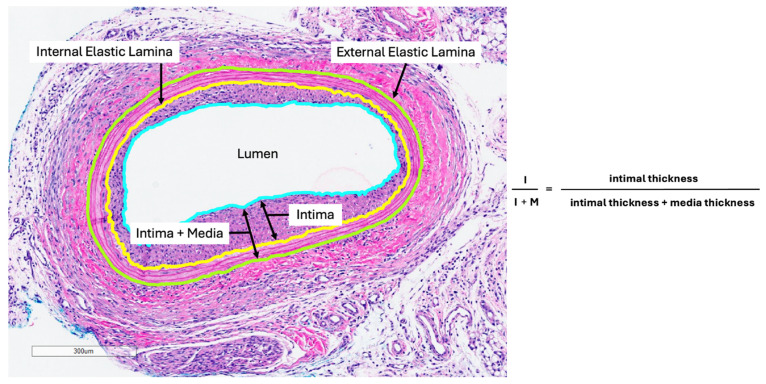
IH measurement in rat CCA post balloon injury. Representative hematoxylin and eosin-stained cross-section of a rat CCA illustrating vessel wall architecture and quantitative measurements used for evaluation of neointimal formation. The lumen is outlined to demonstrate luminal area, while the internal elastic lamina (IEL) and external elastic lamina (EEL) demarcate the boundaries of the intimal and medial compartments. The normal intima is a monolayer of endothelial cells lining the vessel lumen, separated from the media by the IEL. The media consists of VSMCs and elastic tissue, separated from the adventitia by the EEL. The adventitia is the outermost layer, which attaches the vessel to the surrounding connective tissue. Neointimal thickness is measured between the lumen and the IEL, and medial thickness is defined between the IEL and EEL. The intima-to-media ratio is calculated as shown and serves as a standardized metric for quantifying the severity of IH. Abbreviations: EEL: External Elastic Lamina; IEL: Internal Elastic Lamina.

VSMC proliferation and IH development typically peak 2 weeks after surgery [29]. Therefore, vessels are typically perfusion-fixed and explanted 2 weeks after injury [27,28,29,32]. The artery is then embedded in paraffin or optimal cutting temperature (OCT) compound to measure IH [23,25,28,29,32]. The lumen, internal elastic lamina (IEL), external elastic lamina (EEL), intima, and media are defined, and the intima/media ratio is calculated (Figure 2) [27,32]. This morphometric endpoint provides a robust quantitative assessment of restenotic burden and remains the gold-standard readout for therapeutic efficacy in this model.

A variety of techniques can be used to further analyze these tissues to answer different questions, including reendothelialization, inflammation, SMC phenotype, extracellular matrix content, and apoptosis. Techniques used include immunohistochemical staining, the Immunofluorescent TUNEL procedure, and electron microscopy. Some studies looking at different markers are summarized in Table 2.

### 2.5. Advantages and Limitations

The widespread use and translational relevance of the rat carotid artery balloon injury model stem from its reproducibility and clinical relevance. Most notably, the model is highly reproducible, allowing precise control over the extent, location, and severity of vascular injury and remodeling response [25,26]. Balloon size, balloon type (compliant vs. noncompliant), inflation pressure, and the number of passes can be standardized across experiments, resulting in consistent endothelial denudation and medial injury [26]. Additionally, the reproducibility of the lesion permits accurate and reproducible histomorphometric quantification of IH, widely accepted as a gold-standard measure of restenotic burden [26]. This reproducibility enables reliable comparison between experimental groups and facilitates rigorous testing of pharmacologic and genetic interventions [25,26]. Additionally, a strong advantage of this model is the maintenance of normal arterial flow in comparison to ligation models [29]. Mechanistically, the model uses tools similar to those in the clinical setting and closely mimics the mechanical and biological injury induced by angioplasty, including endothelial removal, medial disruption, platelet adhesion, and subsequent IH, thereby capturing the injury-driven processes underlying post-angioplasty restenosis [26,32]. Collectively, these advantageous features establish the rat carotid balloon injury model as a reproducible and translationally relevant tool for investigating the gross morphological, cellular, biochemical, and molecular components of the response to arterial injury.

Despite its widespread use, the rat carotid balloon injury model has several important limitations that must be considered. The procedure is technically demanding, and the reproducibility of vascular injury across animals depends on surgical expertise, familiarity with the procedure, strict adherence to standardized technique, and careful control of operative variables [23,29,31]. Technical constraints related to catheter length further necessitate carotid artery segments of sufficient length, often requiring ligation of distal blood vessels of the ECA to maintain hemostasis, resulting in permanent disruption of blood flow [29]. A major translational limitation of this model is that balloon injury is commonly performed in a healthy, eutrophic artery lacking pre-existing atherosclerotic or vasoproliferative disease, whereas clinical balloon angioplasty is performed in diseased human vasculature [26,32]. Although the response of healthy vessels to balloon injury involves many of the same cellular and molecular pathways activated in diseased vessels, these processes are not biologically equivalent. When the rat carotid balloon injury model is performed in healthy tissue, the adaptive response to injury is driven almost exclusively by VSMCs, whereas in human vasculature, which is usually diseased, a complex and dynamic interplay among VSMCs, endothelial cells, macrophages, and T lymphocytes governs the injury response [26]. Additional anatomical constraints in rats, including a lower percentage of medial wall elastin, a condensed subintimal layer, and the absence of a vasa vasorum further limit direct extrapolation to human disease [26]. Nevertheless, despite these inherent limitations, the rat carotid artery balloon injury model remains a valuable and well-validated tool for studying mechanisms of vascular injury and repair.

## 3. Carotid Artery Ligation Model

### 3.1. Background

The carotid artery ligation model is used to investigate the vascular remodeling response to chronic flow cessation and low shear stress, key stimuli for arterial remodeling and neointimal formation, independent of direct endothelial denudation [27,33,34,39]. Unlike the carotid artery balloon injury model, which induces restenosis through acute mechanical disruption of the vessel wall, carotid artery ligation causes sustained alterations in hemodynamics, leading to VSMC dedifferentiation, proliferation, migration, and extracellular matrix remodeling [35]. This model, therefore, provides a complementary approach for studying IH driven primarily by hemodynamic forces rather than mechanical injury. Understanding the cellular basis of this process is crucial to developing treatments for conditions such as atherosclerosis and restenosis following surgical intervention [34].

### 3.2. Surgical Technique

Although multiple species are used, C57BL/6 is widely used in the carotid artery ligation model. These laboratory mice are commonly used in the study of PAD and IH models and tolerate this surgery well [35]. Mice are genetically well characterized and can be genetically manipulated to introduce transgenes or to disrupt endogenous gene expression, making them an attractive species with which to study the molecular mechanisms that contribute to VSMC proliferation and remodeling [34].

**Figure 3:**
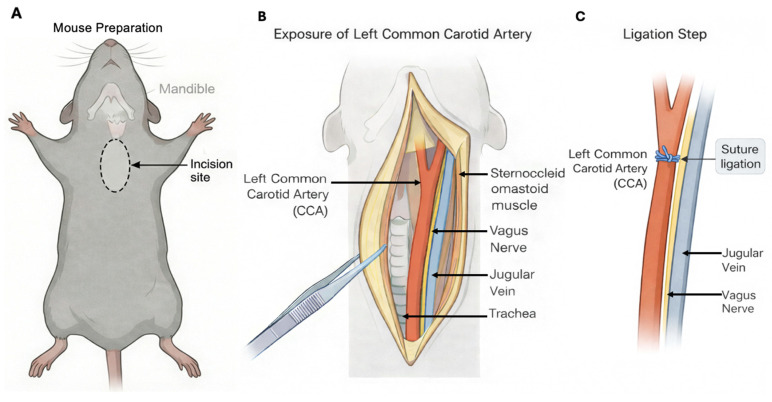
Unilateral left carotid artery ligation model. (**A**) Schematic representation of animal preparation and surgical exposure. Animals are anesthetized and positioned supine, followed by a midline ventral neck incision and retraction to expose the carotid vasculature on the left. (**B**) Illustration of vessel identification and isolation. The left common carotid artery (CCA) is carefully dissected free from surrounding tissues using blunt dissection while preserving adjacent structures, including the jugular vein and vagus nerve. Dissection is carried out distally to expose the carotid artery bifurcation. (**C**) Just proximal to the carotid artery bifurcation, the CCA is ligated to produce cessation of blood flow, thereby inducing flow-mediated vascular remodeling and neointimal formation. Abbreviations: CCA: Common Carotid Artery.

The surgery begins with the mouse lying supine after induction and then maintained in a surgical plane of anesthesia using isoflurane (Figure 3). The fur covering the neck from sternum to chin is removed and the skin is cleaned with alternating betadine swabs and alcohol pads. Surgery is performed using a dissecting microscope or surgical loupes for magnification. A midline neck incision is made, and retraction is brought to the left side and lateral to the trachea. The left salivary gland is moved further laterally by blunt dissection. The pretracheal strap muscles are retracted medially. Blunt dissection is performed using a microdissecting forceps with curved tips to expose the CCA and separate from the vagus nerve. The left CCA is isolated and ligated just proximal to the carotid bifurcation with a 7-0 Prolene suture, resulting in complete disruption of antegrade blood flow (Figure 3) [34]. After ligation and assuring hemostasis, the incision is closed using a 4-0 Vicryl suture.

### 3.3. Pathophysiology

Carotid artery ligation induces vascular remodeling through chronic cessation of blood flow and sustained reduction in shear stress [34,40]. Wall shear stress is the force on endothelial cells, directly proportional to flow velocity and inversely proportional to the vessel radius [40]. Thus, the cessation of forward blood flow secondary to carotid artery ligation causes low shear stress proximal to the ligature [34,40]. In an adaptive attempt to maintain a constant level of shear stress, the vessel undergoes constrictive remodeling, characterized by progressive reduction in luminal diameter [24,40]. This compensatory luminal narrowing depends on an intact endothelium, as endothelial cells act as primary sensors of altered shear stress and respond through mechanotransduction pathways involving the glycocalyx, ion channels, calcium influx, and altered gene transcription [34,37,40].

The marked reduction or cessation of blood flow not only reduces shear stress but also produces regions of tissue hypoxia [35,40,41]. Hypoxia is a primary inflammatory trigger, inducing endothelial cell activation, increased endothelial intracellular calcium, and the production of inflammatory mediators [41]. Activated endothelial cells upregulate leukocyte adhesion molecules, including vascular adhesion molecule-1, intercellular adhesion molecule-1, E-selectin, and P-selectin, which are essential for the recruitment and firm adhesion of leukocytes [24,34,41]. Leukocyte recruitment occurs early, with evidence of leukocyte recruitment detected around the lumen and in the adventitia 3 days post-ligation [24]. Concurrent chemokine production establishes a chemotactic gradient that further promotes the influx of inflammatory cells [34].

Blood stasis, together with turbulence just proximal to the ligating suture, induces delayed thrombosis by first promoting platelet activation [24,41]. Activated platelets form P-selectin-dependent aggregates with leukocytes, which adhere to the endothelium and further enhance the stasis and hypoxia-induced inflammatory response [41]. The high concentration of inflammatory mediators, tissue factors, and platelet phospholipids at the endothelial surface triggers blood coagulation, thereby forming a fibrin matrix along the lumen [41]. This fibrin matrix is later used as a temporary scaffold by migrating VSMCs during neointima formation [41].

An increase in early inflammatory cytokines occurs in parallel with the downregulation of signaling molecules and transcription factors that drive the expression of VSMC contractile proteins [35]. Thus, early inflammatory cytokine signaling drives VSMC dedifferentiation from a contractile to a synthetic, proliferative state [35]. VSMC phenotypic modulation is further regulated by noncoding RNAs, including significant downregulation of microRNAs that normally promote the differentiated state of VSMCs and suppress proliferation and migration [35]. Therefore, the downregulation of these microRNAs has been shown to decrease the expression of VSMC contractile proteins and promote VSMC proliferation and migration [35].

Upregulation of VSMC proliferation and migration, inflammatory cell infiltration, thrombosis, and extracellular matrix production are components of the reparative response to injury that ultimately result in the formation of the neointima [34,40]. However, neointimal development is spatially heterogeneous [34]. Regions near the aortic arch are relatively protected because oscillations of blood flow increase shear stress, whereas the distal segments of the ligated vessel exhibit near-stasis conditions, promoting increased vascular remodeling [34]. The accumulation of the VSMC-rich neointima in combination with the constrictive remodeling secondary to reduced shear stress results in significant, fixed luminal narrowing secondary to carotid artery ligation [34].

### 3.4. Experimental Endpoints

Typically, four weeks after ligation, perfusion fixation is performed, the left and right carotid arteries are excised, fixed, and embedded in paraffin or OCT to allow histological and morphological evaluation [24,27,34]. Alternatively, after excision, the left and right carotid arteries are flash-frozen for DNA, RNA, and/or protein analysis [27].

To perform a morphometric analysis of vascular lesion formation, the location of analysis is crucial, as the severity of the lesion is greatest in the 1 mm closest to the ligature and decreases with distance from the ligature [27,33]. As a result, various models of quantification have been used [27]. These measurements are then used to generate an average neointimal lesion size per mouse [27]. Additionally, the lumen, IEL, EEL, intima, and media are determined and used to calculate the intima/media ratio (Figure 2) [27,33]. Together, these measurements distinguish inward (constrictive) remodeling from neointimal growth [33,34,42].

A variety of techniques, including immunohistochemical staining, qPCR, Western blot analysis, and genome-wide analysis of RNA may be used. This testing allows further investigation of VSMC proliferation, VSMC dedifferentiation, endothelial cell distribution, expression of mRNA encoding transcription factors and smooth muscle contractile proteins, signaling pathways required to sustain a differentiated contractile phenotype, and the inflammatory response [34,35]. Select studies examining different markers are summarized in Table 2.

### 3.5. Advantages and Limitations

The carotid artery ligation model is a widely used and informative platform for studying the effects of chronic alterations in blood flow and shear stress on vascular remodeling and VSMC proliferation. Most notably, the model yields a highly reproducible, consistent, and predictable localized vascular remodeling response when performed using standardized techniques due to its technical simplicity and ease of operation [33,39]. Specifically, the model reliably produces both constrictive remodeling and robust IH, thereby enabling simultaneous study of the geometric and cellular contributors to luminal narrowing at the molecular level [34,42]. As a result of its reproducibility, it serves as an excellent model for comparative and mechanistic studies [33,39].

A key advantage of the carotid ligation model is the preservation of endothelial integrity [34]. Unlike the balloon injury model, the carotid ligation model does not require mechanical trauma and endothelial denudation to induce VSMC proliferation [34]. With the endothelium intact, this model enables the study of endothelium-dependent responses governing inward remodeling and IH, including adhesion molecule expression, inflammatory signaling, and shear-responsive gene regulation [34,40]. The model further contributes to defining the roles of adhesion molecules, inflammatory mediators, signaling pathways, and noncoding RNAs in vascular remodeling by using transgenic and knockout mice, which are amenable to genetic and molecular manipulation [34,35,41]. Additionally, the ligation limits systemic inflammatory responses, thereby enabling interpretation of vessel-specific outcomes. For example, this model is particularly valuable for defining causal mechanisms of VSMC phenotypic switching, proliferation, and migration.

Despite its utility for studying flow-dependent vascular remodeling, the carotid artery ligation model has several important limitations that should be considered when interpreting experimental findings and translating results to human disease. Most notable is the potentially limited clinical and pathophysiological relevance of this model. Specifically, ligating the CCA induces acute, near-complete flow cessation, which often exaggerates the partial stenosis, complex flow disturbances, or heterogeneous shear-stress patterns commonly observed in human vascular disease [37,40]. Thus, the mechanisms responsible for vascular remodeling and neointimal formation in the carotid artery ligation model may differ from those involved in the physiologic injury setting, limiting direct clinical extrapolation [34]. Additionally, the physiologic and translational relevance is further limited by the fact that the ligation is commonly performed in a healthy, non-atherosclerotic artery, rather than a diseased vessel [34,39]. A healthy vessel is fundamentally different from a diseased vessel, preventing the model from studying key aspects of advanced atherosclerosis, including lipid-rich necrotic cores, fibrous caps, calcification, or plaque rupture [39]. Further, key anatomical differences exist between murine and human carotid anatomy [34]. Specifically, murine carotid arteries lack a well-developed vasa vasorum and exhibit different elastic properties compared with human arteries, which may influence remodeling responses and limit translational applicability [34]. It is for these reasons that further studies are required to clarify the translational relevance of the carotid artery ligation model to specific clinical scenarios of vascular disease and restenosis in humans [34].

Another important limitation of this model involves data analysis. Commonly, inflammation-dependent activation of coagulation and luminal fibrin formation can occur following ligation, leading to thrombus formation near the ligation site [41]. This clot formation at the suture site prevents reliable analysis of the artery [33]. To overcome the unreliable analysis secondary to thrombus formation, many studies reduce the usable sample size by excluding arterial segments within approximately 1 mm of the ligature from analysis [33]. Additionally, the carotid artery ligation model often displays heterogeneity in remodeling, with the magnitude of neointimal formation greatest near the ligature and progressively diminishing along the length of the artery [33]. This heterogeneity causes challenges in data analysis and interpretation, requiring standardization of section location and distance from the ligature, which ultimately increases technical and analytical complexity [33].

## 4. Partial Carotid Artery Ligation Model

### 4.1. Background

Similar to the carotid artery ligation model, the partial carotid artery ligation model was developed to investigate vascular remodeling and IH induced by disturbed blood flow, a key hemodynamic determinant of vascular disease [37]. However, unlike complete CCA ligation, which induces complete flow cessation, the partial carotid artery ligation model selectively ligates the ICA, the ECA and specific branches of the ECA, leaving a single small branch open. This model results in a marked reduction in blood flow, while maintaining vessel patency [36]. This model of disturbed flow is further characterized by low and oscillatory wall shear stress, hemodynamic conditions that closely resemble those observed at arterial bifurcations, aortic arches, and other regions predisposed to atherosclerosis [36,37]. These disturbed flow environments promote endothelial dysfunction, inflammatory activation, and VSMC proliferation, ultimately leading to neointimal formation and progressive luminal narrowing [37,38]. Thus, the partial carotid artery ligation model is relevant for studying the mechanistic relationship between disturbed flow with low and oscillatory shear stress and vascular pathology, as well as for testing therapeutic interventions targeting flow-mediated vascular disease [37].

### 4.2. Surgical Technique

The partial carotid artery ligation model often utilizes C57B1/6 and ApoE^-/-^ mice to study how disturbed blood flow and shear stress induce atherosclerosis in a predisposed genetic model [38]. It is widely accepted that mutations in the ApoE gene in humans cause familial hypercholesterolemia, which ultimately leads to atherosclerosis [38]. Therefore, ApoE^-/-^ mice have been developed to study the pathogenesis of atherosclerosis [38].

**Figure 4:**
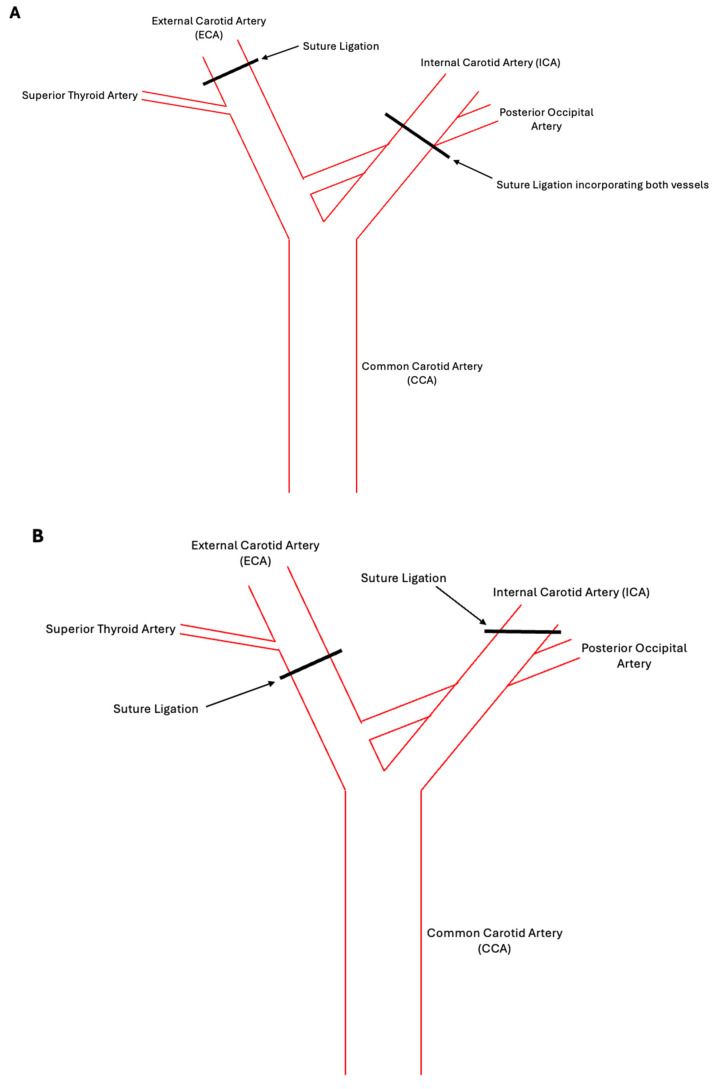
Partial ligation—a mouse model of IH. (**A**) Mouse partial carotid ligation model with patent superior thyroid artery. Schematic illustration of the partial ligation configuration used to induce disturbed flow in the left common carotid artery (CCA). The external carotid artery (ECA) is ligated distally. A single ligature is placed to simultaneously occlude the internal carotid artery (ICA) and posterior occipital artery, while the superior thyroid artery remains patent. This ligation strategy produces marked reductions in flow and altered shear stress patterns within the left CCA, while preserving limited outflow through the superior thyroid artery. (**B**) Mouse partial carotid ligation model with patent posterior occipital artery. Schematic representation of the partial ligation configuration used to generate disturbed flow in the left CCA. The ECA, ICA, and superior thyroid artery are ligated, while the posterior occipital artery remains patent. This ligation strategy reduces carotid blood flow and induces low, oscillatory shear stress within the CCA, while preserving limited outflow through the posterior occipital artery. Abbreviations: CCA: Common carotid artery; ECA: External Carotid Artery; ICA: Internal Carotid Artery.

The surgery begins with the mouse lying supine after induction and then maintained in a surgical plane of anesthesia using isoflurane. The fur covering the neck from sternum to chin is removed and the skin is cleaned with alternating betadine swabs and alcohol pads. Surgery is performed using a dissecting microscope or surgical loupes for magnification. A ventral midline neck incision is made, and the left CCA is exposed by blunt dissection [37]. The CCA, ICA and ECA are dissected out. To develop a model of flow-induced vascular remodeling, once the left CCA is isolated, blood flow in the left CCA is partially reduced due to ligation of the ICA and ECA with the outflow to a single branch vessel being the superior thyroid artery or the occipital artery [36]. To do so, two different surgical techniques may be performed. The first technique involves ligating the ECA (away from the origin) and the ICA near its origin, incorporating the occipital artery, while the superior thyroid artery is left patent (Figure 4A) [37]. In the second technique, the ECA (proximal to the superior thyroid artery) and the ICA (away from its origin) are ligated, thus maintaining flow through the superior thyroid artery (Figure 4B) [36]. Ligations are performed with 6-0 silk suture. The incision is then be closed with 4-0 Vicryl suture.

### 4.3. Pathophysiology

Partial carotid artery ligation induces vascular remodeling and atherosclerosis through reduced blood flow, characterized by low and oscillatory wall shear stress, key stimuli for endothelial dysfunction and lesion formation [37]. These hemodynamic alterations in the low-flow ligated artery initiate time-dependent structural vascular remodeling, including lumen narrowing, medial thickening, and intimal formation [36].

Exposure to low and oscillatory shear stress induces endothelial dysfunction, characterized by the downregulation of atheroprotective genes and activation of proatherogenic mediators and proinflammatory pathways [37]. Additionally, endothelial dysfunction leads to increased generation of reactive oxygen species (ROS), which contributes to endothelial injury and negative vascular remodeling [37]. Once inflammatory cells infiltrate the developing plaque, they produce pro-inflammatory cytokines that amplify local inflammatory signaling and further promote lesion progression [38]. In summary, reduced blood flow, in combination with an inflammatory and oxidative stress environment, stimulates VSMC proliferation, migration, and extracellular matrix production, leading to progressive intima-media thickening and vessel wall remodeling [36].

A direct link exists between disturbed flow and atherogenesis [37,38]. This relationship is exemplified in studies where partial carotid ligation is performed on ApoE-deficient mice, leading to rapid atherosclerotic plaque formation [37,38]. Collectively, the partial carotid artery ligation model reproduces a sequential cascade in which disturbed flow induces endothelial dysfunction, inflammatory cell activation and infiltration, vascular remodeling, and ultimately accelerated plaque formation.

### 4.4. Experimental Endpoints

A high-resolution Doppler ultrasound is used to measure flow velocity, vessel dimensions, and vessel length at the inlet, midpoint, and outlet of the mouse common carotid arteries before and after partial ligation [37,38]. These measurements are then used in computational fluid dynamics (CFD) modeling to estimate shear stress magnitudes and directions to further confirm the low and oscillatory wall shear stress in the ligated artery [37].

Two days to six weeks after partial carotid artery ligation, perfusion fixation is performed; the left and right carotid arteries are excised, fixed, and embedded in paraffin or OCT to allow histological and morphological evaluation [36,37,38]. The size of the atherosclerotic plaque is quantified using histological measurements of plaque area [38]. Additionally, the lumen, IEL, EEL, intima, and media are defined and used to calculate the intima/media ratio (Figure 2) [36]. Further, lipid deposition, indicative of atherosclerotic lesion development, is also analyzed [38].

The localization and proliferation rates of inflammatory cells can be measured [36]. Specifically, inflammatory cells are identified by immunohistochemistry, using the common leukocyte antigen, CD45 marker [36]. Immunohistochemistry is also performed to detect monocytes and/or macrophages using the CD14 marker and M1 macrophages using the CD11c marker [38]. To further assess the pro-inflammatory immune response, the immunohistochemical markers TNF-α and IL-6 may be used [38]. Immunohistochemistry can also be used to measure protein levels for ICAM-1 and VCAM-1 as an endothelial marker [37]. Additionally, at the molecular level, isolation of intimal RNA from carotid arteries enables mechanistic studies of endothelial gene expression using qPCR [20].

### 4.5. Advantages and Limitations

The key advantage of the partial carotid artery ligation model is its physiologic relevance to human disease, specifically as a model of flow-mediated atherosclerosis [37]. The partial carotid artery ligation model produces low and oscillatory shear stress, closely replicating the disturbed-flow environments of regions prone to atherosclerosis in human arteries [37]. Additionally, maintaining patency through residual branches, such as the superior thyroid artery or the occipital artery, enables the study of vascular remodeling under physiologic pressure and flow regulation [36]. As a result, this model induces vascular remodeling that closely resembles structural changes in human disease, including intima–media thickening and compensatory outward remodeling [36]. Thus, this model provides valuable, translationally relevant insight into the mechanisms linking altered hemodynamics to human cardiovascular disease [36].

In addition to being translationally relevant, the model is highly reproducible, displaying consistent survival rates and predictable remodeling responses following ligation [36]. The vascular remodeling response also occurs along the entire length of the artery, enabling accurate morphometric analysis and reproducible quantification of remodeling parameters [36].

Another key advantage of the partial carotid artery ligation model is the preservation of endothelial integrity. Not only does the presence of an intact endothelium allow the investigation of endothelial mechanotransduction, inflammatory activation, and gene expression changes in response to disturbed shear stress, but it also reduces the risk of thrombosis often seen in complete occlusion models [36].

The partial carotid artery ligation model also effectively causes accelerated atherosclerotic lesion formation, including the development of advanced plaque features such as cholesterol clefts and intraplaque neovascularization, within several weeks [37]. As a result, partial carotid artery ligation enables the investigation of early and advanced stages of atherosclerosis within a shorter timeframe, making it an effective model for studying disease progression [37].

Despite its utility for studying disrupted flow-mediated vascular remodeling and atherosclerosis, the partial carotid artery ligation model has several important limitations that should be considered when interpreting experimental findings. A major limitation that the partial carotid artery ligation model shares with the carotid artery ligation model is the use of healthy murine arteries lacking pre-existing atherosclerotic disease. Another shared limitation of the carotid artery ligation and partial carotid artery ligation models is species-specific anatomical differences, including variations in arterial wall composition and absence of a well-developed vasa vasorum, which may influence remodeling response and limit direct translational applicability.

Another limitation is that consistent branch ligation and reproducible flow reduction depends on precise surgical technique. Variability in the degree of residual flow can lead to differences in shear stress magnitude and remodeling responses across animals, potentially introducing experimental variability.

Additionally, the acute hemodynamic alteration caused by partial ligation differs from the gradual development of arterial stenosis and chronic flow disturbances typically seen in human vascular disease. Therefore, early molecular responses may reflect acute adaptive signaling in addition to long-term pathogenic mechanisms.

## 5. Hindlimb Ischemia Model

### 5.1. Background

PAD is a major cause of morbidity and mortality worldwide, affecting over 230 million people globally [43]. Additionally, with increasing life expectancy, the number of patients with PAD is expected to grow [44]. PAD is characterized by progressive occlusion of peripheral arteries leading to impaired tissue perfusion, claudication, and critical limb ischemia [44]. Experimental animal models of hindlimb ischemia are therefore essential for investigating the cellular and molecular mechanisms of vascular remodeling and for evaluating novel therapeutic approaches to improve blood flow to the lower extremities and promote vessel growth [44]. Among these, the murine hindlimb ischemia (HLI) model has become the most widely used platform due to its reliability and reproducibility [44]. In the HLI model, the occlusion of the femoral artery results in neovascularization, specifically angiogenesis and arteriogenesis, to restore perfusion [44]. Arteriogenesis is the formation of collateral arteries from the pre-existing arteriolar network, while angiogenesis is the process of forming new capillaries from the pre-existing microvasculature [44,45]. The murine HLI model provides an efficient, translationally relevant platform for testing pharmacologic, genetic, and cell-based interventions to enhance vascular regeneration in ischemic tissues [44,46]. Additionally, the availability of numerous transgenic mouse strains enables mechanistic investigation of signaling pathways involved in inflammation, endothelial activation, and vascular growth, further strengthening the value of this model in cardiovascular research [44].

### 5.2. Surgical Technique

**Figure 5:**
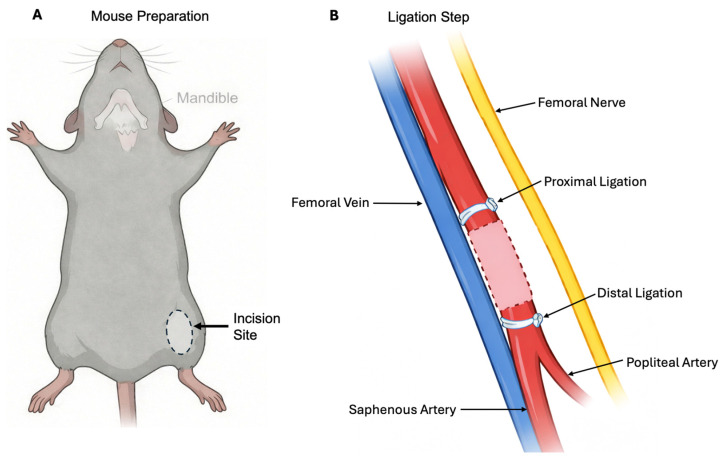
Mouse hindlimb ligation ischemia model. (**A**) Schematic illustration of the unilateral hindlimb ligation procedure. (**B**) A proximal ligature is placed along the femoral artery near the inguinal ligament to reduce arterial inflow, followed by a distal ligature positioned just proximal to the superficial caudal epigastric artery branch. This configuration produces a reproducible reduction in limb perfusion and establishes a defined ischemic territory for experimental analysis.

The hindlimb ischemia model commonly utilizes C57BL/6 mice, as they demonstrate fast perfusion recovery after femoral artery ligation [44]. Hindlimb ischemia is typically induced through surgical ligation and excision of a portion of the femoral artery and its major branches, producing a controlled reduction in limb perfusion. The surgery begins with the mouse lying supine after induction and then maintained in a surgical plane of anesthesia using isoflurane. Hair is removed from the operative limb, and the area is sanitized with alcohol pads and betadine swabs [47]. Surgery is performed using a dissecting microscope at 10× or 20× magnification, or surgical loupes [47]. An approximately 1 cm longitudinal incision is made from approximately 7 mm below the inguinal region and approximately 3 mm above it [46,47]. The subcutaneous fat tissue surrounding the thigh muscle is pushed away using a PBS-moistened fine pointed cotton swab and forceps [46,47]. The cautery is then applied transversely to incise and dissect through the subcutaneous fat tissue to reveal the femoral artery [47]. Retractors are used to obtain a better view of the lower extremity vasculature [47]. Blunt dissection is performed using micro forceps and a fine pointed cotton swab to expose the neurovascular bundle [47]. The femoral artery is then dissected and separated from the femoral vein and nerve proximally and distally [46,47]. The femoral artery is then ligated proximally, as close to the inguinal ligament as possible, and distally, just proximal to the branch of the superficial caudal epigastric artery, using 7-0 silk sutures (Figure 5) [45,46,47]. Transect and excise the segment of the femoral artery between the proximal and distal ligations [46,47]. Branch vessels, including the profunda femoris and superficial epigastric arteries, may also be ligated depending on the desired severity of ischemia. After confirmation of hemostasis, the incision is closed using 5-0 Vicryl sutures [47].

Alternative surgical approaches have been described to produce graded levels of ischemia, including single ligation of the femoral or iliac artery, complete excision of the femoral artery and its side branches, electrocoagulation of the common iliac artery and/or femoral artery, or ameriod constrictors allowing investigators to tailor the model to the experimental question being studied [46]. Regardless of the specific technique used, the contralateral limb is typically left untreated and serves as an internal control for subsequent perfusion and histologic analyses [48].

### 5.3. Pathophysiology

Surgical interruption of the femoral artery produces an immediate reduction in limb perfusion, initiating a cascade of ischemia-driven cellular and molecular responses aimed at restoring tissue blood supply. Acute ischemia results in tissue hypoxia, metabolic stress, and accumulation of ROS, which together stimulate activation of endothelial cells, inflammatory cell recruitment, and release of cytokines and growth factors that regulate vascular remodeling [44,46,49,50].

The two principal adaptive processes that occur after ligation are arteriogenesis and angiogenesis. Arteriogenesis is the enlargement and remodeling of pre-existing collateral arterioles in the proximal limb and is driven primarily by increased shear stress within collateral vessels following arterial occlusion [45,46,48]. Shear stress-mediated endothelial activation causes collateral vessel maturation by promoting the expression of adhesion molecules and chemokines that recruit monocytes and macrophages, which in turn release growth factors such as vascular endothelial growth factor (VEGF), fibroblast growth factor (FGF), and platelet-derived growth factor (PDGF) [44,51,52].

In parallel, angiogenesis occurs predominantly in the distal ischemic skeletal muscle, where tissue hypoxia induces stabilization of hypoxia-inducible factor-1α (HIF-1α), leading to transcriptional activation of proangiogenic genes including VEGF and stromal cell-derived factor-1 (SDF-1) [46,52]. These signaling pathways promote endothelial cell proliferation, migration, and capillary sprouting, resulting in the formation of new microvascular networks that improve oxygen delivery to ischemic tissue [44,52].

Over time, the combined effects of arteriogenesis, angiogenesis, and skeletal muscle regeneration lead to gradual recovery of perfusion, although the extent and rate of recovery vary depending on genetic background, age, and comorbid conditions [44,48]. Collectively, this hindlimb ischemia model reproduces the key biological processes underlying ischemia-induced vascular repair, providing a mechanistic platform for studying therapeutic strategies that enhance neovascularization and tissue regeneration.

### 5.4. Experimental Endpoints

The principle functional endpoint for the HLI models is recovery of limb perfusion over time, which is most commonly assessed using laser Doppler perfusion imaging (LDPI) to measure relative blood flow between the ischemia and contralateral control limb [44,47,48]. Serial blood flow measurements using LDPI are generally performed preoperatively and at predetermined time points postoperatively, including immediately after the surgery, 3 and 7 days after the surgery, and then weekly for 2 to 4 weeks [44,48].

Animals are sacrificed at a predetermined time point [48]. Our lab sacrifices the animals 14 days after the surgery. In order to perform morphological and histological analysis, the gastrocnemius, adductor magnus, semimembranosus, and semitendinosus are fixed and embedded in paraffin or OCT [48]. Alternatively, the portion of the tissue used for RNA extraction and qRT-PCR gene expression is snap frozen.

Histological and immunohistochemical analyses are used to quantify capillary density, collateral vessel formation, and arteriolar remodeling within ischemic tissues [44]. Capillary density is typically evaluated with immunohistochemical staining for endothelial cells using the markers CD31 and Von Willebrand Factor (Table 3) [44,48]. Further, proliferative activity is measured [48]. Angiogenesis and arteriogenesis are assessed by immunohistochemical staining using antibodies against α-smooth muscle actin and VEGF (Table 3) [44,48]. Additionally, T-lymphocytes can be identified using the CD3 marker, and macrophages were identified using the F4/80 marker (Table 3) [48].

### 5.5. Advantages and Limitations

The murine HLI model remains one of the most widely utilized experimental platforms for studying PAD due to its reproducibility, technical flexibility, and strong pathophysiologic relevance. Surgical interruption of the femoral artery produces a predictable reduction in limb perfusion followed by progressive vascular remodeling, thereby enabling controlled investigation of endogenous neovascularization mechanisms [48]. The model reliably induces both arteriogenesis in proximal collateral vessels and angiogenesis within distal skeletal muscle, allowing investigators to distinguish between complementary vascular growth processes that contribute to perfusion recovery [44,46].

A major strength of the HLI model is its experimental versatility. Modifications in ligation strategy, arterial excision, or branch vessel manipulation permit graded severities of ischemia, allowing studies to be tailored to specific mechanistic or therapeutic questions [44]. The availability of numerous transgenic mouse strains further enables targeted investigation of molecular pathways regulating endothelial function, inflammation, and vascular growth, significantly enhancing mechanistic resolution [44].

Importantly, the HLI model exhibits robust translational relevance, as perfusion recovery depends on collateral vessel development and capillary remodeling processes analogous to those observed in human PAD [48]. Serial perfusion imaging techniques, particularly laser Doppler perfusion imaging, provide quantitative, noninvasive assessment of blood flow recovery over time, strengthening the model’s utility for evaluating therapeutic interventions [48]. Additionally, use of the contralateral limb as an internal control reduces inter-animal variability and improves statistical power [48].

Despite its widespread use, this hindlimb ischemia model has several limitations that must be considered when interpreting experimental outcomes. One limitation is the acute nature of surgically induced ischemia, which differs from the chronic, progressive arterial narrowing characteristic of human PAD [44]. The abrupt reduction in blood flow produces injury and remodeling responses that may not fully replicate the gradual evolution of a chronic process [46].

Another important limitation is the frequent use of young, healthy animals lacking comorbid conditions such as diabetes, hyperlipidemia, or advanced age, all of which strongly influence vascular remodeling and perfusion recovery in humans [44]. These biological differences can lead to exaggerated regenerative responses and limit direct clinical extrapolation [48]. Furthermore, substantial strain-dependent variability has been documented, with different murine genetic backgrounds demonstrating markedly divergent perfusion recovery kinetics and collateral formation capacity [44].

Variability in surgical technique, including the extent of arterial excision, branch ligation, and perioperative tissue handling, can significantly alter ischemic severity and remodeling responses [48]. Additionally, murine hindlimbs exhibit robust collateralization capacity relative to humans, which may attenuate ischemic injury and influence therapeutic effect [48].

While this model is highly effective for studying neovascularization, it incompletely reproduces certain features of advanced human disease, including atherosclerotic plaque burden, chronic inflammation, and long-standing microvascular dysfunction, thereby limiting translational implications [44,46].

## 6. Ischemia–Reperfusion Injury Model

### 6.1. Background

Ischemia–reperfusion injury (IRI) is a is an ongoing challenge, occurring when restoration of blood flow exacerbates cellular injury following a period of ischemia. This phenomenon has significant clinical relevance in a variety of pathologies, including arterial embolization, arterial thrombosis, vascular trauma, advanced peripheral vascular disease, traumatic crush injuries, and/or prolonged tourniquet application [56,57]. The pathological consequences of reperfusion arise from a complex interplay of oxidative stress, endothelial dysfunction, inflammatory activation, and mitochondrial injury [56,58].

Experimental models were developed to investigate the acute inflammatory, immune reaction, and pathophysiological response to limb IRI [54]. Among these approaches, tourniquet-based techniques using elastic or rubber bands have become widely adopted due to their technical simplicity, reproducibility, and ability to produce rapid and reversible ischemia without direct vascular surgery [55]. Importantly, these models mimic clinically relevant scenarios such as tourniquet application or acute vascular occlusion followed by revascularization, both of which are known to generate substantial reperfusion-mediated injury [56,57].

### 6.2. Surgical Technique

**Figure 6:**
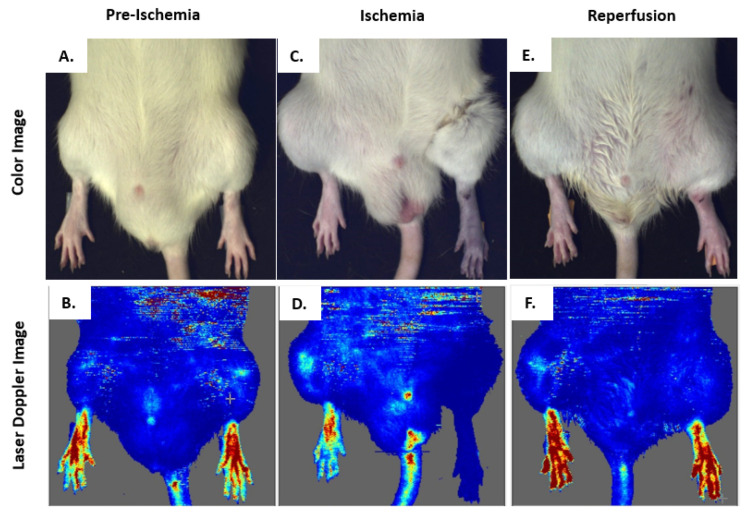
Hindlimb ischemia–reperfusion injury (IRI) model and monitoring by gross appearance and laser Doppler perfusion imaging [56]. Representative images obtained before ischemia (**A**,**B**), during ischemia (**C**,**D**), and following reperfusion (**E**,**F**). Color photographs (**A**,**C**,**E**) demonstrate gross changes in limb coloration associated with arterial occlusion and subsequent reflow. Corresponding laser Doppler perfusion imaging maps (**B**,**D**,**F**) illustrate perfusion dynamics, with a marked reduction in blood flow during ischemia and partial restoration upon reperfusion. Warmer colors indicate higher perfusion, whereas cooler colors represent reduced tissue blood flow. Adapted with permission from [56].

Animals are induced and anesthetized with isoflurane to maintain a surgical plane of anesthesia. Atropine is given prior to the procedure to prevent excessive salivation. An orthodontic rubber band is placed around the left proximal thigh for 2 h to produce acute unilateral hindlimb ischemia [53]. Successful induction of ischemia is typically confirmed by pallor of the limb and marked reduction in distal perfusion, often monitored using LDPI (Figure 6). After 2 h (range up to 6 h), the orthodontic rubber band tourniquet is released to restore blood flow. Reperfusion is visually confirmed by restoration of limb coloration and functionally by recovery of LDPI-detected blood flow.

### 6.3. Pathophysiology

The pathophysiology of limb IRI is initiated by acute interruption of blood flow, resulting in tissue hypoxia, depletion of intracellular ATP, metabolic acidosis, and accumulation of ischemic metabolites [56,57]. However, the most severe local and systemic harm occurs during reperfusion rather than ischemia itself. Reintroduction of oxygen leads to excessive ROS generation, which drives lipid peroxidation, protein oxidation, DNA damage, and mitochondrial dysfunction [56,58].

Mitochondrial injury plays a central role in IRI, as impaired electron transport chain activity amplifies oxidative stress and promotes activation of apoptotic and oxidative stress pathways [56]. Reperfusion also leads to endothelial dysfunction and inflammatory signaling, causing leukocyte adhesion, neutrophil infiltration, and cytokine release [56,58]. Activated inflammatory cells exacerbate tissue injury by releasing proteases and additional ROS, leading to microvascular dysfunction, capillary leakage, and tissue edema [56,57].

Although blood flow is restored, these processes result in skeletal muscle necrosis and impaired microvascular perfusion, a defining feature of reperfusion injury [56,57].

### 6.4. Experimental Endpoints

A primary functional endpoint of the model is the assessment of skeletal muscle blood flow during ischemia and following reperfusion, most commonly measured using LDPI (Figure 6) [55].

At a predetermined time post-injury (24 h in our lab), the animals are sacrificed and blood is collected by cardiac puncture for serum analysis, and the lung, kidney, liver, gastrocnemius muscle (ischemic limb and control limb), and tibialis anterior muscle (ischemic limb and control limb) are collected for either histological analysis or for RNA extraction and qPCR gene expression [53,54]. The portion of the tissue used for morphological and histological analysis is fixed and embedded [54]. Alternatively, the portion of the tissue used for RNA extraction and qRT-PCR gene expression is snap frozen [54].

Histological evaluation by quantifying and grading skeletal muscle injury, including myofiber degeneration, interstitial edema, inflammatory cell infiltration, and tissue necrosis, is performed to determine the magnitude of injury [53,54]. Leukocyte infiltration in skeletal muscles and remote organs is also measured [54]. To assess for kidney and liver injury, serum blood urea nitrogen (BUN), creatinine (Cr), alanine aminotransferase (ALT), aspartate aminotransferase (AST), and albumin levels are analyzed [54].

Immunohistochemical examination is used to determine the degree of apoptosis in skeletal muscle by evaluating the expression of caspase 3 [53]. Additionally, inflammatory markers may be measured (Table 3) [54]. Total RNA from remote organs and muscles is extracted, converted to cDNA, and analyzed by RT-qPCR to assess gene expression patterns and molecular networks associated with early immune cell activation and inflammation [54]. Given the central role of oxidative injury in IRI, qRT-PCR and Western blot analysis are frequently used to measure oxidative stress (Table 3) [53,55]. Additionally, mitochondrial dysfunction represents a defining feature of reperfusion injury and is commonly evaluated through assays of electron transport chain activity and metabolic enzyme function [55].

### 6.5. Advantages and Limitations

The rubber band hindlimb IRI model offers several advantages. It is technically simple, rapid, and minimally invasive, avoiding direct vascular surgery while producing reproducible ischemia [55]. The model provides precise temporal control of ischemia and reperfusion, a critical feature for studying early reperfusion events and oxidative injury mechanisms. Additionally, this approach closely mimics clinically relevant conditions such as tourniquet-induced ischemia and surgical revascularization [57]. The murine platform further allows integration of transgenic models for mechanistic investigation of inflammatory and oxidative stress pathways [58].

Despite its strengths, several limitations exist. The model induces acute ischemia, which differs from the chronic and progressive perfusion deficits characteristic of many human vascular diseases [57]. Variability in ischemia severity may occur depending on rubber band tension and placement [55]. Furthermore, the model produces global limb ischemia rather than focal arterial occlusion, potentially altering microvascular responses. Species-specific differences in metabolism, inflammatory signaling, and collateral circulation also constrain direct clinical extrapolation [58].

## 7. Conclusions

Experimental animal models of arterial disease remain indispensable for advancing the understanding of the mechanistic basis of cardiovascular disease, vascular remodeling, and ischemic tissue injury. Additionally, these models are crucial in pharmacological advancements. Rodent models, in particular, are the most commonly used, as they accurately simulate characteristics of human cardiovascular disease, including endothelial dysfunction, IH, adaptive vascular remodeling, inflammation, and oxidative stress, while maintaining strong reproducibility and genetic modifiability. However, it is important to note that no single rodent model fully encapsulates the complexity of human arterial disease; rather, each model offers specific advantages for studying defined pathophysiologic mechanisms. Accordingly, appropriate model selection should be guided by the specific biological processes and careful consideration of the strengths and limitations of each model (Table 4).

## Figures and Tables

**Table 1:** Genetic, dietary, and pharmacological rodent models of arterial disease.

Model/Genotype	Species/Strain	Vascular Phenotype	Diet/Induction	Primary Use
Atherosclerosis Models				
ApoE−/− mouse [7,8,9]	Mouse/C57BL/6J	Spontaneous hypercholesterolemia and atherosclerotic lesions; endothelial dysfunction	Standard chow	Gold-standard atherosclerosis model; plaque biology; anti-atherosclerotic drug testing
ApoE−/− + Western diet (diet-augmented protocol) [7,8]	Mouse/C57BL/6J	Accelerated hypercholesterolemia and atherosclerosis vs. standard chow alone; increased lesion area and macrophage infiltration	Western diet: 21% fat, 0.15–0.2% cholesterol	Most commonly used accelerated atherosclerosis protocol; anti-atherosclerotic drug and genetic intervention testing
LDLR−/− mouse [7,9,10]	Mouse/C57BL/6J	Minimal atherosclerotic lesions and moderate hypercholesterolemia on standard chow diet; accelerated robust atherosclerosis and hypercholesterolemia on high-fat diet; elevated LDL	Western diet: 21% fat, 0.15–0.2% cholesterol; Paigen diet: 15% fat, 1.25% cholesterol, 0.5% cholic acid	LDL-centric atherosclerosis; cholesterol-lowering therapy; combined metabolic syndrome models
ApoE−/− rat [2,3,11]	Rat/Sprague-Dawley or Wistar	Atherogenic phenotype; hypercholesterolemia; hyperlipidemia	Standard chow; Western diet: 21.3% fat, 1.25% cholesterol	Atherosclerosis model; larger vessel size enables catheter-based and surgical interventions not feasible in mice
Diabetes-Associated Vascular Disease Models				
db/db mouse (Leprdb/db) [12,13,14]	Mouse/C57BL/KSJ	Hyperglycemia; hyperinsulinemia; robust insulin resistance; hyperphagia; obesity; hypertrophic vascular remodeling; endothelial dysfunction	Standard chow; high-carbohydrate cafeteria diet	Genetic model of obesity and T2DM; T2DM macrovascular and microvascular disease; diabetic cardiomyopathy; endothelial dysfunction
STZ-induced diabetic ApoE−/− mouse [6,15,16,17]	Mouse/C57BL/6J or CD-1	Hyperglycemia; atherosclerosis; accelerated aortic plaques vs. ApoE−/− alone; increased lesion complexity; endothelial dysfunction	STZ induction (40–55 mg/kg/day × 5 days IP); standard chow	T1DM model; Diabetes-accelerated atherosclerosis; T1DM vascular complications; hyperglycemia-specific pathway testing; evaluate therapeutic options
Zucker Diabetic Fatty (ZDF) rat (fa/fa) [6,18]	Rat/Zucker Fatty	Insulin resistant; hyperinsulinemia; early onset diabetes; hyperlipidemia, obesity; aortic stiffness; impaired endothelial function;	Standard rat chow	Well-established model of T2DM and obesity; T2DM-associated microvascular complications and diabetic nephropathy; insulin sensitizing agent testing; anti-inflammatory and vasoregulatory drug testing
ApoE−/−; db/db mouse (double mutant) [6,19]	Mouse/C57BL/6J or C57BLKS/J	Robust dyslipidemia; severe hypercholesterolemia; hyperglycemia; T2DM; accelerated atherosclerosis vs. ApoE-/- alone	Standard chow	T2DM model; reproduces mechanistic link between T2DM and atherosclerosis; cardiometabolic drug testing
Hypertension Models				
Spontaneously Hypertensive Rat (SHR) [5]	Rat/Wistar	Progressive hypertension; cardiomyocyte hypertrophy; coronary artery smooth muscle cell hypertrophy; inward vascular remodeling; increased wall-to-lumen ratio; arterial stiffness;	Standard rat chow	Gold-standard hypertension model; antihypertensive drug testing; hypertensive vascular remodeling
Dahl Salt-Sensitive (SS) rat [20]	Rat/Sprague-Dawley	Salt-dependent hypertension; central arterial stiffness preceding BP elevation; endothelial dysfunction; vascular remodeling; renal damage	High-salt diet (4% NaCl)	Salt-sensitive hypertension; dietary sodium–vascular interaction; arterial stiffness; renal-vascular coupling

Abbreviations: ApoE, apolipoprotein E; BP, blood pressure; db/db, leptin receptor-deficient (Leprdb/db); IP, intraperitoneal; LDLR, low-density lipoprotein receptor; SHR, spontaneously hypertensive rat; SS, Dahl salt-sensitive; STZ, streptozotocin; T1DM, type 1 diabetes mellitus; T2DM, type 2 diabetes mellitus; ZDF, Zucker diabetic fatty.

**Table 2:** Experimental endpoints in common rodent models of intimal hyperplasia.

	Mechanism of Injury	Primary Outcome	Tissue/Cell Markers	Cellular Health	ECM Health	Inflammatory Markers	Inflammatory Cell Markers
Carotid Artery Balloon Injury	Mechanical denudation of ECs, Stretch-induced VSMC activation	Intimal Hyperplasia [27,32]	CD31, SMCα [27,29]	PCNA [29], TUNEL [27]	PSR, Alcian Blue [29]	iNOS^+^, MHC II^+^, IL-1β, TNF-α [29]	CD3, CD11b CD45, CD68^+^, F4/80 [27,29]
Carotid Artery Ligation	Ischemia, loss of flow, platelet adhesion	Intimal Hyperplasia [27,33]	SMCα, Von Willebrand Factor [34], Myh11 Acta2, Tagln, Spp1, Vinculin [35]	BrdU labeling–replication index [34], mRNA of SMC contractile proteins, mRNA of transcription factors [35]		IL-1β, IL-6, TNF-α, Chemokine ligand 2, secreted phosphoprotein 1 [35]	CD45, Antibodies to monocytes and macrophages [34]
Partial Carotid Artery Ligation	Low flow plus oscillatory stress	Intimal Hyperplasia [36], CFD [37]		Oil Red O stain [38]		IL-6, TNF-α [38], ICAM-1, VCAM-1, PECAM-1 [37]	CD45 [36], CD14, CD11c [38]

Abbreviations: Acta2, actin alpha 2; BrdU, bromodeoxyuridine; CD3, cluster of differentiation 3; CD11b, cluster of differentiation 11b; CD11c, cluster of differentiation 11c; CD14, cluster of differentiation 14; CD31, cluster of differentiation 31; CD45, cluster of differentiation 45; CFD, computational fluid dynamics; ICAM-1, intercellular adhesion molecule-1; IL-1β, interleukin-1 beta; IL-6, interleukin-6; iNOS, inducible nitric oxide synthase; Myh11, myosin heavy chain 11; PCNA, proliferating cell nuclear antigen; PECAM-1, platelet endothelial cell adhesion molecule-1; PSR, Picosirius Red; SMCα, smooth muscle cell-α; Spp1, secreted phosphoprotein-1; Tagln, transgelin; TNFα, tumor necrosis factor alpha; TUNEL, terminal deoxynucleotidyl transferase; VCAM-1, vascular cell adhesion molecule-1.

**Table 3:** Experimental endpoints in hindlimb ischemia and ischemia–reperfusion injury models.

	Mechanism of Injury	Primary Outcome	Tissue/Cell Markers	Tissue Health	Inflammation Markers	Inflammatory Cell Markers
Hindlimb Ischemia	Ischemia	Blood Flow, Angiogenesis, Arteriogenesis [44,47]	CD31, Factor VII, α-SMA [44,48]	VEGF, BrdU [48]		CD3, F4/80 [48]
Ischemia–Reperfusion Injury	Ischemia + Reperfusion	Blood Flow, myofiber degeneration, interstitial edema, inflammatory cell infiltration, and tissue necrosis [53], leukocyte infiltration [54]	Immune System Cells	Cleaved Caspase-3 [53], serum BUN, Cr, ALT, AST, albumin [54], mitochondrial dysfunction [55]	IL-1β, IL-4, IL-5, IL-6, IL-10, IFN-γ, TNF-α, C-X-C motif chemokine ligand-1, monocyte chemoattractant protein-1 [54], MnSOD, MDA, oxidative stress [55], iNOS [53]	CD68, MPO [54]

Abbreviations: ALT, alanine aminotransferase; AST, aspartate aminotransferase; BrdU, bromodeoxyuridine; BUN, blood urea nitrogen; CD3, cluster of differentiation 3; CD31, cluster of differentiation 31; CD68, cluster of differentiation 68; Cr, creatinine; IFN-γ, interferon gamma; IL-1β, interleukin-1 beta; IL-4, interleukin-4; IL-5, interleukin-5; IL-6, interleukin-6; IL-10, interleukin-10; iNOS, inducible nitric oxide synthase; MDA, malondialdehyde; MnSOD, manganese superoxide dismutase; MPO, myeloperoxidase; VEGF, vascular endothelial growth factor; α-SMA, alpha-smooth muscle actin.

**Table 4:** Cross-model comparison of surgical rodent models for the study of peripheral arterial disease.

Model	Applicable Research Scenarios	Simulated Pathological Features	Clinical Translational Value
Carotid Artery Balloon Injury	Studying mechanisms of post-angioplasty restenosis; evaluating pharmacologic and genetic interventions targeting VSMC proliferation, migration, and neointimal formation; testing therapeutic strategies to prevent restenosis after arterial reconstruction.	Endothelial denudation; medial disruption; platelet adhesion and activation; inflammatory cell recruitment; VSMC dedifferentiation from contractile to synthetic phenotype; VSMC proliferation and migration; extracellular matrix deposition; neointimal formation; progressive luminal narrowing.	Closely mimics the mechanical and biological injury induced by balloon angioplasty, including endothelial removal, medial disruption, and subsequent IH. Performed in a healthy artery without pre-existing atherosclerotic disease, limiting direct extrapolation to diseased human vasculature. Rat anatomy differs from human arteries in elastin content, subintimal structure, and absence of vasa vasorum.
Carotid Artery Ligation	Studying flow-mediated vascular remodeling and IH independent of mechanical injury; investigating roles of hemodynamic forces, shear stress, endothelial mechanotransduction, and VSMC phenotypic switching; defining molecular mechanisms of inflammatory signaling using transgenic and knockout mouse models.	Near-complete flow cessation; low shear stress; inward (constrictive) remodeling; endothelial activation; leukocyte recruitment; VSMC dedifferentiation; VSMC proliferation and migration; extracellular matrix remodeling; fibrin matrix formation; neointimal formation; luminal narrowing.	Models the hemodynamic consequences of flow cessation and low shear stress relevant to post-surgical arterial remodeling. However, the acute and near-complete occlusion does not replicate the partial stenosis or complex flow patterns typical of human disease. Performed in healthy, non-atherosclerotic arteries, limiting study of advanced atherosclerotic features. Murine anatomy differs from human carotid arteries.
Partial Carotid Artery Ligation	Studying the mechanistic relationship between disturbed flow, low and oscillatory shear stress, endothelial dysfunction, and atherosclerosis; investigating early and advanced stages of plaque formation; testing therapeutic interventions targeting flow-mediated vascular disease, particularly in atherosclerosis-prone genetic models (e.g., ApoE−/− mice).	Reduced blood flow with preserved vessel patency; low and oscillatory wall shear stress; endothelial dysfunction; downregulation of atheroprotective genes; increased ROS generation; inflammatory cell recruitment; VSMC proliferation and migration; intima-media thickening; accelerated atherosclerotic plaque formation including advanced features (cholesterol clefts, intraplaque neovascularization).	Closely replicates disturbed-flow environments at arterial bifurcations and regions predisposed to atherosclerosis in humans. Maintains vessel patency, preserving physiologic pressure and flow regulation. However, the acute induction of disturbed flow differs from the gradual development of arterial stenosis in human disease. Performed in healthy murine arteries lacking pre-existing atherosclerosis; species-specific anatomical differences limit direct translational applicability.
Hindlimb Ischemia	Studying mechanisms of ischemia-induced neovascularization (angiogenesis and arteriogenesis); evaluating pharmacologic, genetic, and cell-based interventions to enhance vascular regeneration; investigating molecular pathways regulating endothelial function, inflammation, and vascular growth in the setting of peripheral limb ischemia.	Acute reduction in limb perfusion; tissue hypoxia and metabolic stress; ROS accumulation; endothelial cell activation; inflammatory cell recruitment; arteriogenesis; angiogenesis; skeletal muscle regeneration; gradual perfusion recovery.	Replicates key biological processes underlying ischemia-induced vascular repair analogous to those in human PAD, including collateral vessel development and capillary remodeling. Serial LDPI enables quantitative, noninvasive perfusion monitoring. However, surgical ischemia is acute rather than progressive, unlike chronic PAD. Performed in young, healthy animals lacking comorbidities. Murine hindlimbs have greater collateralization capacity than humans, potentially attenuating ischemic injury. Does not reproduce atherosclerotic plaque burden or chronic microvascular dysfunction.
Ischemia–Reperfusion Injury	Studying the acute inflammatory, oxidative, and mitochondrial injury responses triggered by reperfusion following ischemia; investigating mechanisms of leukocyte adhesion, neutrophil infiltration, cytokine release, and remote organ injury; evaluating pharmacologic interventions targeting oxidative stress and inflammatory pathways in the setting of IRI.	Acute limb ischemia followed by reperfusion; tissue hypoxia and ATP depletion; metabolic acidosis; excessive ROS generation upon reperfusion; lipid peroxidation; mitochondrial dysfunction; endothelial dysfunction; leukocyte adhesion and neutrophil infiltration; cytokine release; microvascular dysfunction; capillary leakage; tissue edema; skeletal muscle necrosis.	Closely mimics clinically relevant scenarios including tourniquet-induced ischemia and surgical revascularization followed by reperfusion injury. Technically simple and reproducible without direct vascular surgery. However, the model induces acute, global limb ischemia rather than focal arterial occlusion, which differs from focal reperfusion injury in clinical settings. Species-specific differences in metabolism, inflammatory signaling, and collateral circulation limit direct extrapolation to human disease.

Abbreviations: ApoE, apolipoprotein E; IH, intimal hyperplasia; LDPI, laser Doppler perfusion imaging; PAD, peripheral arterial disease; ROS, reactive oxygen species; VSMC, vascular smooth muscle cell.

## Data Availability

This article is a review article and original data is not presented.

## Abbreviations

The following abbreviations are used in this manuscript:

ALT	Alanine Aminotransferase
AST	Aspartate Aminotransferase
BUN	Blood Urea Nitrogen
CCA	Common Carotid Artery
CFD	Computational Fluid Dynamics
Cr	Creatinine
ECA	External Carotid Artery
EEL	External Elastic Lamina
FGF	Fibroblast Growth Factor
HIF-1	Hypoxia Inducible Factor-1α
HLI	Hindlimb Ischemia
ICA	Internal Carotid Artery
IEL	Internal Elastic Lamina
IFN-γ	Interferon-γ
IH	Intimal Hyperplasia
iNOS	Inducible Nitric Oxide Synthase
LDPI	Laser Doppler Perfusion Imaging
MDA	Malondialdehyde
MnSOD	Manganese Superoxide Dismutase
MPO	Myeloperoxidase
OCT	Optimal Cutting Temperature
PAD	Peripheral Arterial Disease
PDGF	Platelet-Derived Growth Factor
PSR	Picosirius Red
ROS	Reactive Oxygen Species
SDF-1	Stromal Cell-Derived Factor-1
TNF-α	Tumor Necrosis Factor-α
VEGF	Vascular Endothelial Growth Factor
VSMC	Vascular Smooth Muscle Cell
